# Prevalence and factors associated with pregnancy-induced hypertension in public general hospitals, central zone, Tigray Region Ethiopia: a cross-sectional study

**DOI:** 10.11604/pamj.2026.54.23.23847

**Published:** 2026-06-02

**Authors:** Ebud Ayele Dagnazgi, Kinfe Tsehaye, Kidane Zereabruk, Teklewoiyni Mariye, Tsgehana Gebregyorgis, Tsiyon Brhanu, Woldu Aberhe, Teklemariam Gebregziabher, Teklehaimanot Gereziher Haile, Shewit Engdashet Berhe

**Affiliations:** 1 Department of Public Health Nutrition, College of Medicine and Health Sciences, Axum University, Tigray, Ethiopia,; 2 Department of General Public Health, College of Medicine and Health Sciences, Axum University, Ethiopia,; 3 Department of Adult Health Nursing, School of Nursing, Aksum University, Aksum, Ethiopia,; 4 Department of Epidemiology and Biostatistics, College of Health Sciences, Aksum University, Aksum, Ethiopia,; 5 School of Nursing, College of Health Sciences, Axum University, Tigray, Ethiopia

**Keywords:** Ethiopia, pregnancy-induced hypertension, Tigray Region

## Abstract

**Introduction:**

hypertensive disorders represent the most common medical complications in pregnancy and the leading cause of poor prenatal outcomes both in developed and developing countries. Although many studies have been done elsewhere in the globe, there is limited data on pregnancy-induced hypertension disorders and their associated factors in resource-limited Ethiopia, especially in the Tigray Region's Central Zone. Therefore, this study was designed to assess the prevalence of pregnancy-induced hypertension and its associated factors among pregnant women in General Hospitals, Central Zone, Tigray Region, Ethiopia.

**Methods:**

a hospital-based retrospective “cross-sectional” study design was conducted on 769 selected women who delivered at the General Hospitals of Central Zone from August 8, 2018 to July 7, 2019. A systematic sampling technique was used to select the study participants. The collected data were entered using Epi-Data 3.02; exported to and analyzed by Statistical Package for Social Science version 22. Bivariate and multivariable analysis was employed to assess the relationship between independent and dependent variables. The odds ratio along with 95% CI was estimated to identify factors associated with the outcome variable. The level of significance was declared at P-value ≤ 0.05.

**Results:**

seven hundred sixty-nine (100%) documents were included in the study. The prevalence of pregnancy-induced hypertension was found to be 5.1% (95% CI, 3.5%-6.6%). It was more likely to occur in mothers who had previous hypertension [(AOR=7.16, 95% CI: (1.65, 43.97)], substance abuse [(AOR=7.25, 95% CI: (1.43, 36.73)], family history of pregnancy-induced hypertension [(AOR=5.43, 95%: CI: (1.16, 25.45)], and multiple pregnancy [(AOR=37.09, 95% CI: (8.70, 158.06)].

**Conclusion:**

pregnant women who had previous hypertension, substance abuse, family history of pregnancy-induced hypertension, and multiple pregnancies had a significant association with pregnancy-induced hypertension in this study.

## Introduction

Pregnancy-induced hypertension is a high blood pressure and one of the leading causes of mortality and morbidity among pregnant women around the globe today [1]. It is characterized by either blood pressure levels of 140/90 mm Hg or higher after 20 weeks of gestation, or a blood pressure rise greater than 30/15 from early. Such mild forms of pregnancy-induced hypertension require close monitoring. The severe forms of pregnancy-induced hypertension are reflected through blood pressure levels of 160/100 taken on two separate occasions, 6 hours apart, after bed rest [2]. About 350,000 women die every year from pregnancy-related causes globally and more than half of deaths occur in sub-Saharan Africa (SSA), and approximately 12% of maternal deaths are associated with pregnancy-induced hypertension (PIH) disorders. Pregnancy-induced hypertension complicates 10% of all pregnancies; around 40,000 women, mostly from developing countries, die each year due to preeclampsia or eclampsia [3]. Hypertensive disorder of pregnancy is associated with adverse maternal and prenatal outcomes increased maternal mortality, increased risk of cerebrovascular accidents, renal failure, and pulmonary edema, increased risk of placental abruption [4].

Pregnancy-induced hypertension is associated with adverse maternal and prenatal outcomes increased maternal mortality, increased risk of cerebrovascular accidents, renal failure, and pulmonary edema, and increased risk of placental abruption [4,5]. Maternal mortality in Ethiopia due to pregnancy-induced hypertension between 1980 and 2012 increased from 4%-29% at different health facilities [6]. And preeclampsia contributed to the complication of approximately 1% of all deliveries and 5% of all pregnancies. Moreover, 16% of direct maternal mortality and 10% of indirect maternal mortality were due to preeclampsia/eclampsia [7]. Even though many studies have been done elsewhere in the world, there is limited data on the prevalence of pregnancy-induced hypertension and its associated factors in resource-poor countries like Ethiopia, especially in Tigray Region. This study aimed to determine the prevalence and associated factors of pregnancy-induced hypertension, so providing information that exists on the ground has paramount importance to the Central Zone General Hospital, district health offices, patients, and health care professionals who are working in hospitals. Also, this study raises awareness among healthcare professionals about the prevalence of hypertension and contributing factors. Pregnant women who are at risk of developing pregnancy-induced hypertension also benefited indirectly. In addition, the information generated from this study helps as a source of information for other researchers to conduct similar studies in different study areas.

## Methods

**Study design and settings:** a retrospective cross-sectional study design was conducted in public general hospitals of Ethiopia, Tigray, and Central Zone from August 8, 2018 to July 7, 2019. Central Zone of Tigray is one of the seven zones of Tigray Region, which contains three general hospitals called Abyi-Adi, Aksum, and Adwa General Hospital. Based on the 2007 census conducted by the Central Statistical Agency of Ethiopia, the total population was 1,245,824, of which 14.2% were living in the urban area. According to this census, 613,797 were men and 632,027 were women.

**Study population:** all pregnant women who were delivered at the public general hospital of the Central Zone were the source population. Whereas the study population was all selected pregnant women who were delivered in public general hospitals in the Central Zone. Women who were incompetent their document was excluded whereas. The required sample size was determined using the single population proportion formula with the assumption by taking prevalence of previous study done in Mizan-Tip pregnancy-induced hypertension 7.9% [8], 95% level of confidence and 2% marginal error. By adding 10% contingency, the sample size was 769. Cards for each mother were selected using a systematic random sampling technique. The sample size was distributed into each hospital in accordance with the magnitude of the population.

**Data collection:** after providing three days of training to the data collectors five percent pretested was conducted in Shire general hospital to check the completeness and consistency of the developed form and revision and edition takes place. The data was collected by three trained BSc midwives and one BSc midwife as supervisor for a total of ten days. The required data were filled from the selected charts by using a structured data entry format prepared for this purpose. Information about patients' demographic data, past medical history, past obstetric history and laboratory investigations was collected. The required data which was missed from the client's chart was searched and filled into the data entry format from registration books, anesthesia room registrations and operation log books. Meanwhile the completeness of the data was checked every day by the principal investigator and supervisor. Regular supervision and follow-up were made by principal investigator. In addition, regular checkup for completeness and consistency of the data was made on a daily basis.

**Operational definition:**

**Pregnancy-induced hypertension disorder:** hypertension with or without proteinuria or edema two readings of diastolic blood pressure 90/110 mmHg, 4-6 hours apart, which develops after 20 weeks of gestation [4].

**Mild pre-eclampsia:** two readings of diastolic blood pressure 90-109mmHg, 4-6 hours apart, after 20 weeks of gestation and with proteinuria of 300mg/dl in 24 hours or up to 2+ and with/without edema [4].

**Pre-eclampsia:** diastolic blood pressure is equal to or greater than 110mmHg after 20 weeks of gestation. Associated with severe headache, blurred vision, epigastric pain, hyper-reflexia, oliguria, proteinuria (protein equal or greater than 5g/24 hours; dipstick +) and the patient is conscious [9].

**Eclampsia** is signs and symptoms of severe preeclampsia and convulsions or coma [9].

**Statistical analysis:** the data collected from the charts was edited and entered, by Epi-Data version 3.02. Then it was exported and analyzed by SPSS version 22. Data cleaning was performed to check for frequencies, accuracy, and consistencies and missed values and variables. Any error identified during data entry was corrected after revision of the original completed data entry format. All the data obtained from the study population was entered, cleaned and analyzed by the investigator. To explain the study population in relation to relevant variables, descriptive statistics were used. Independent variables with a p-value of = 0.25 in univariate analysis were included in the multivariable analysis to control confounding factors. Multicolinearity was checked to see the linear correlation among the independent variables by using standard error. The fitness of the model was tested by Hosmer-Lemeshow's goodness-of-fit test model coefficient with an enter method was found to be insignificant with a large P-value (P=0.05). Adjusted odds ratio along with 95% CI was estimated to identify the factors associated with pregnant-induced hypertension disorder using multivariable logistic regression analysis. Level of statistical significance was declared at a p-value less or equal to 0.05.

**Ethical consideration:** clearance was obtained from the Institutional Health Research Ethical Review Board (IHRERC) of Aksum University College of Health and Medical Science and a formal letter was written to Central Zone general hospitals ensuring the approval of the proposal. All hospital medical directors were permitted to conduct the study. The data were collected by review of the registration books using structured checklists.

## Results

**Socio-demographic characteristics of the respondents:** a total of 769 women who delivered were included in the study with a response rate of 100%. The mean age of the mothers was 36 (SD ± 1.3) years. And seven hundred forty-two (96.5%) of them were married; 424 (55.1%) of them were women urban residents, and about 614 (79.9%) of women were multipara (Table 1).

**Table 1:** socio-demographic profile of study population of a study conducted on magnitude of pregnancy-induced hypertension and associated factors among deliverd women in public hospitals, Tigray, Ethiopia, 2019

Variables	Frequency	Percent
**Age group mother**		
Less than19	42	5.5
20-24	119	15.5
25-29	226	29.4
30-34	243	31.6
Greater than 35	139	18.1
**Child sex**		
Male	300	39.0
Female	469	61.0
**Marital-status**		
Married	742	96.5
Single	27	3.5
**Residence**		
Urban	424	55.1
Rural	345	44.9
**Parity**		
Premi-para	155	20.2
Multi-para	614	79.9

### Maternal health history and health characteristics

Out of 769 about 39(5.1%) were have been pregnancy-induced hypertension; about 713 (92.7%) pregnant women have been single type pregnancy, and 732 (95.2%) women's were haven't history of previous surgery. In addition to that 721 (93.8%) women were haven't history of abortion, and 649 (84.4%) women's were haven't history of low birth weight, and women 725 (94.3%) were haven't previous family history of pregnancy-induced hypertension. About 606 (78.8%) women's were two times antenatal care follow up, about 766 (99.6%) were haven't diabetes mullets, about 736(95.7%) women's were haven't previous history of hypertension, and 726(94.4%) doesn't have been history of cardiovascular disease; about 690 (89.7%) women were haven't renal disease during pregnancy, and 724 (94.1%) women during pregnancy were haven't any substance abuse, about 434(56.4%) women during pregnancy were have planned and wanted pregnancy, and about 705(91.7%) women were haven't family history of diabetes mullets, about 501(65.1%) women were spontaneous vaginal delivery and 660(85.8%) mothers were with optimal nutrition states (Table 2).

**Table 2:** health profile of study population of participants of a study conducted on magnitude of pregnancy-induced hypertension and associated factors among delivered women in public hospitals, Tigray, Ethiopia, 2019

Variable	Category	Frequency	Percent
**Type of pregnancy**			
Single		713	92.7
Multiple		56	7.3
**Maternal previous surgery**			
Yes		37	4.8
No		732	95.2
**History abortion**			
Yes		48	6.2
No		721	93.8
**History Low birth weight**			
Yes		120	15.6
No		649	84.4
**Family history of pregnant induced hypertension**			
Yes		44	5.7
No		725	94.3
**How many anti-natal care**			
One		25	3.3
Two		606	78.8
Three		138	17.9
**Previous history hypertension**			
Yes		33	4.3
No		736	95.7
**Previous diabetes-mellites**			
Yes		3	.4
No		766	99.6
**Dose have cardiovascular disease**			
Yes		43	5.6
No		726	94.4
**Kidney disease**			
Yes		79	10.3
No		690	89.7
**Have hypertension**			
Yes		39	5.1
No		730	94.9
**Type of hypertension**			
Per-eclampsia		16	2.1
Mild-eclampsia		12	1.6
Eclampsia		12	1.6
**Substance abuse**			
Yes		45	5.9
No		724	94.1
**Family history of diabetes-mellites**			
Yes		64	8.3
No		705	91.7
**Pregnancy states**			
wanted		434	56.4
unwanted		335	43.6
**Mid-upper arm circumstance (MUAC)**			
under nutrition		43	5.6
normal nutrition		726	94.4

### Factor associated with pregnancy-induced hypertension

All 769(100%) of the document were included in the study. The prevalence of pregnancy-induced hypertension was found to be 5.1% (95% CI, 3.5%-6.6%). Variables associated in adjusted analysis: previous history of gestational hypertension were more likely [(AOR=7.16, 95% CI: (1.16, 43.9)] to have pregnancy-induced hypertension, substance abuse was more likely [(AOR=7.24, 95% CI: (1.42, 36.7)] to have pregnancy-induced hypertension, family history of hypertension where more likely [(AOR=5.43, 95% CI: (1.16, 25.45)], to have pregnancy-induced hypertension, multiple type of pregnancy where more likely [(AOR=37.09, 95% CI: (8.7, 158.06)] to implement pregnancy-induced hypertension (Table 3).

**Table 3:** logistic regression analysis on association with pregnancy-induced hypertension among deliverd women in public hospitals, Tigray, Ethiopia, 2019

Variables	Pregnant induced hypertension		COR95% C. I	P-value	95% C.I. AOR	p-value
	yes	no				
**Previous history of gestational hypertension**						
Yes	22	11	47.07(20.92,105.88)**	<0.001	7.157(1.17, 43.97) *	.034
No	30	706	1		1	
**Kidney disease**						
Yes	21	58	7.697 (4.16,14.24)**	<0.001	3.81(0.8,18.11)	.93
No	31	659				
**Substance abuse**						
Yes	32	13	86.65 (39.60,189.58) **	<0.001	7.25(1.43, 36.73) *	.017
No	20	704	1			
**Family history of diabetes-mellites**						
Yes	31	33	30.60(15.90,58.90)**	<0.001	1.653(0.34, 8.06)	.534
No	21	684	1		1	
**Pregnancy status**						
Wanted	20	414	1		1	
Unwanted	32	303	2.19 (1.23,3.90)*	0.0087	2.01(0.68, 5.91)	.207
**Family history of Pregnant induced hypertension**						
Yes	16	28	10.94(5.43,22.02) **	<0.001	5.43(1.16, 25.45) *	.032
No	36	689	1		1	
**Type of pregnancy**						
Single	17	696	1		1	
Multiple	35	21	68.24 (33.08,140.75)**	<0.001	37.09(8.70, 158.06) **	.000
**Mid-upper arm circumstance (MUAC)**						
<21cm	20	23	18.86 (9.40,37.83)	<0.001	2.40(0.46, 12.46)	.298
>=21cm	32	694	1		1	
P=<0.001** p<0.05*						

## Discussion

This study assessed the prevalence and factors associated with pregnancy-induced hypertension in public general hospitals in the Central Zone, Tigray Region, Ethiopia. The prevalence of pregnancy-induced hypertension was 5.1% with 95% CI of (3.5%, 6.6%); magnitude of pre-eclampsia, mild eclampsia-, and eclampsia was 16 (2.1%), 12 (1.6%) and 12 (1.6%), respectively. Which is lower than the study conducted in India, was 5.4% while preeclampsia and eclampsia accounts 44% and 40% respectively [10]. In African Americans it accounts 6.4% of deliveries [3]. In Brazil, Assis *et al*. (2008) study 14.5% of pregnant women were diagnosed [11]. India 2006 was 5.38% [10]. A study employed in Jimma Hospital found that the prevalence of hypertensive disorders of pregnancy was 8.5% [12]. Dessie referral hospital is found to be 8.4% [13], Mizan-Tepi university teaching hospital, Tepi and Gebretsadik Shawo hospitals, the prevalence of pregnancy-induced hypertension was (7.9%) [14], Dilla University Referral Hospital was found to be 2.2% [15] and South Africa was 12% [16] respectively. However, this finding is higher than the studies conducted in Nepal 4.4% were diagnosed as pre-eclampsia /eclampsia, with a proportion of 3.9%, 0.4%, 0.1% mild pre-eclampsia, severe pre-eclampsia and eclampsia respectively [17]. Another study indicates that the prevalence of preeclampsia in developing countries ranges from 1.8% to 16.7% [18]. In South Africa from 22,711 deliveries in Dora Nginza Hospital over the two-year period (2007 & 2008), showed that the prevalence of hypertension in pregnancy as 6.7% (66.9 per 1000 deliveries) and the incidence of pre-eclampsia was 35.4% and chronic hypertension 2.8% [19]. Studies in Ethiopia 5% [20]. Study in southern part of Brazil in (2011) gestational hypertension was (4.3%) [21]. The possible reason for this difference might be due to social-demographic characteristics and promotion differences facility to the maternal health. Furthermore, this discrepancy might be also due to difference in lifestyle and safe health service environment.

In this study, mothers with previous hypertension, substance abuse, family history of pregnancy-induced hypertension and type of pregnancy were found to be significantly associated with pregnancy-induced hypertension. In this regard mothers with previous hypertension were 7.16 times more likely [(AOR=7.16, 95% CI: (1.17, 43.97)] to have gestational hypertension than those who hadn't before. This finding is similar to the study done in a different area [22-25]. The reason might be almost mothers with having history of previous induced hypertension can have problems with their life span due to physiological genetic factor.

This study revealed that women with substance abuse were 7.2 times [(AOR=7.25, 95% CI: ( 1.43, 36.73)] to have pregnancy-induced hypertension than their counterparts. This was similar to the systematic review done from different literature [26]. This is because substance abuse causes social, psychological and economical problem for pregnant women's that can lead to increased stress, and also some substances can cause for physiological regulation problems that lead to increased blood pressure in pregnant women. Family history of pregnancy-induced hypertension where 5.43 times more likely [(AOR=5.43, 95% CI: (1.159, 25.45)] to have gestational hypertension than those counterparts. This finding was similar to the study done in Amhara and others [13,22]. This might have occurred due to the genetic factor that can develop for the physiological predisposition to pregnancy-induced hypertension to their offsprings.

The study showed that mothers with multiple types of pregnancy were 37.09 times more likely [(AOR=37.09, 95%: (8.70, 158.06)] to have gestational hypertension than single type pregnancy. This finding was similar to the study done in Dessia referral hospital [27]. This is because of mothers having exposure towards multiple pregnancy can deferrer in physiological and mental states compares to the single type pregnancy.

**Strengths of this study:** even though this study was conducted from secondary data resources. Information about patients' demographic data, past medical history, past obstetric history and laboratory investigations were collected. The required data which was missed from the client's chart was searched and filled into the data entry format from registration books, anesthesia room registrations and operation log books. This study also didn't expose to recalling bias.

**Weakness of the study:** the study was conducted in three health facility, and this affects the representativeness of the study results. Involving more health facilities at different levels could have produced better generalizable results. Our study is a retrospective study based on secondary data, so it was not possible to determine all factors such as occupation, educational level, and economic status of pregnant women were missed that could affect pregnant-induced hypertension.

## Conclusion

This study indicated that the prevalence of pregnancy-induced hypertension was slightly lower, and those pregnant women with previous hypertension, substance abuse, family history of pregnancy-induced hypertension and multiple pregnancies had significant association with pregnancy-induced hypertension. Based on the finding of this study the following recommendations are forwarded: attendance of anti-natal care should give special focus on early treatment and prevention for those women's having family history pregnancy-induced hypertension, pregnant women with previous history pregnancy-induced hypertension and multiple pregnancies. Community health workers, anti-natal care attendance should work on the behavioral change of substance abuse women.

***What is known about this topic***

- *Pregnancy-induced hypertension is still a health problem concern in the study area;*
- *There was lack of information on pregnancy induced hypertension in the study area;*
- *Even though pregnancy-induced hypertension is preventable through promoting behavioral change, the prevalence is still increasing dramatically*.

***What this study adds***

- *Pregnancy-induced hypertension in the study area was found about one from twenty pregnant women;*
- *Pregnant women with previous hypertension, substance abuse, family history of pregnancy-induced hypertension and multiple pregnancies were the significant factors of pregnancy-induced hypertension;*
- *Based on the finding, health care professionals should work on the prevention and behavioral change actions before any complication development on mothers by information, education and communication approach*.
